# Supplementary material for: Effects of Ethanol on Expression of Coding and Noncoding RNAs in Murine Neuroblastoma Neuro2a Cells
Source: Int J Mol Sci. 2022 Jun 30;23(13):7294. doi: 10.3390/ijms23137294 (PMC9267046; doi:10.3390/ijms23137294)
Supplement: Supplementary file 1 [file ijms-23-07294-s001.zip › Figure S1.pptx]

## Slide 1
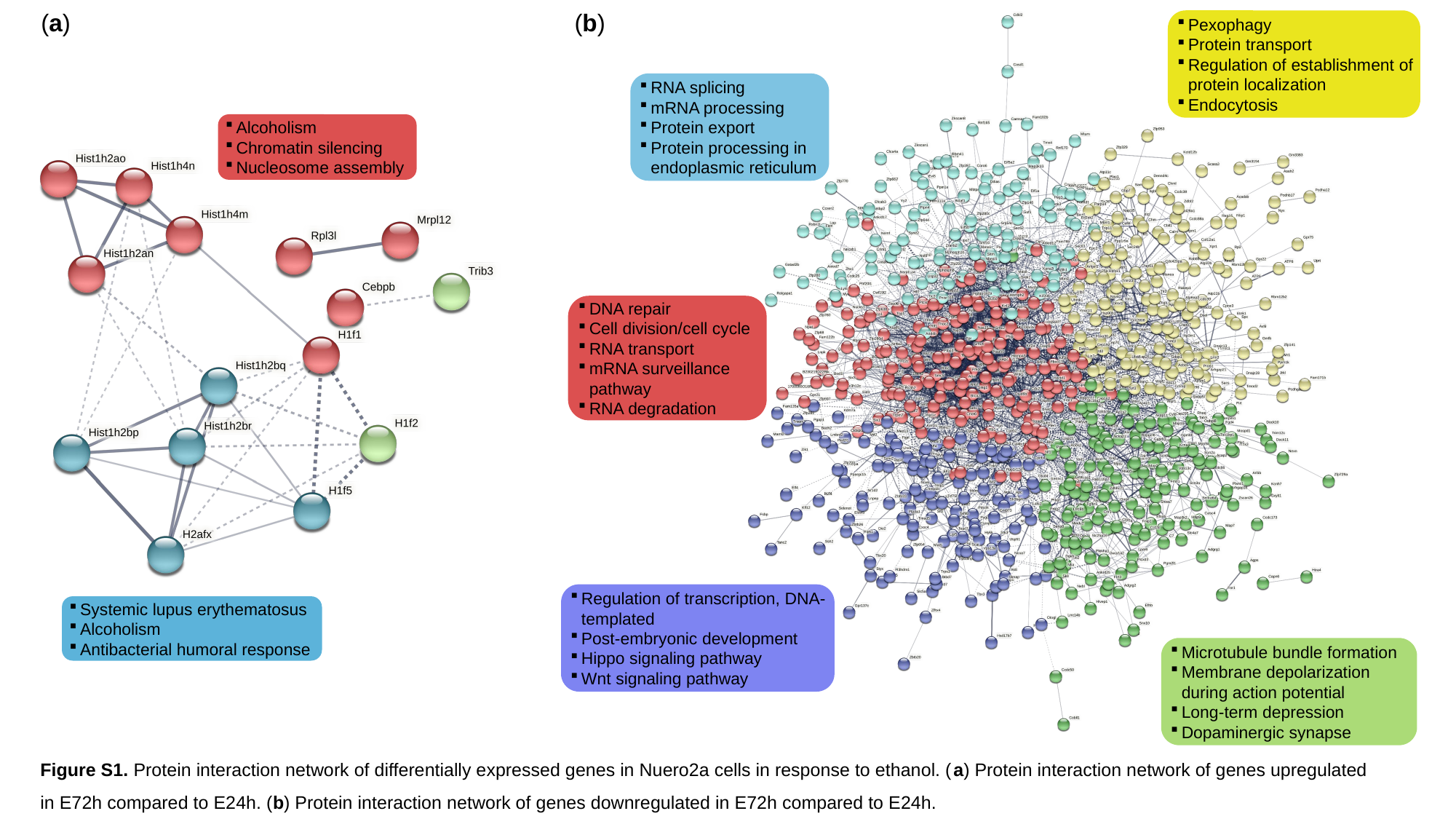

(a)
(b)
Pexophagy
Protein transport
Regulation of establishment of protein localization
Endocytosis
RNA splicing
mRNA processing
Protein export
Protein processing in endoplasmic reticulum
DNA repair
Cell division/cell cycle
RNA transport
mRNA surveillance pathway
RNA degradation
Regulation of transcription, DNA-templated
Post-embryonic development
Hippo signaling pathway
Wnt signaling pathway
Microtubule bundle formation
Membrane depolarization during action potential
Long-term depression
Dopaminergic synapse
Alcoholism
Chromatin silencing
Nucleosome assembly
Systemic lupus erythematosus
Alcoholism
Antibacterial humoral response
Figure S1. Protein interaction network of differentially expressed genes in Nuero2a cells in response to ethanol. (a) Protein interaction network of genes upregulated in E72h compared to E24h. (b) Protein interaction network of genes downregulated in E72h compared to E24h.
