# Supplementary material for: Effects of Ethanol on Expression of Coding and Noncoding RNAs in Murine Neuroblastoma Neuro2a Cells
Source: Int J Mol Sci. 2022 Jun 30;23(13):7294. doi: 10.3390/ijms23137294 (PMC9267046; doi:10.3390/ijms23137294)
Supplement: Supplementary file 1 [file ijms-23-07294-s001.zip › Table S2.pdf]

Table S2. Primers used in RT-qPCR

Gene	Forward (5′-3′)	Reverse (5′-3′)	Amplicon size (bp)
Gapdh	AGGTCGGTGTGAACGGATTTG	TGTAGACCATGTAGTTGAGGTCA	123
Atm	CCAGCTTTTTGATGCAGATACCA	CTTCCCAGCCTACGTCTATTTTC	119
Bmpr2	TTGGGATAGGTGAGAGTCGAAT	TGTTTCACAAGATTGATGTCCCC	115
Rb1	TCGATACCAGTACCAAGGTTGA	ACACGTCCGTTCTAATTTGCTG	95
Hes7	CGGGAGCGAGCTGAGAATAG	CACGGCGAACTCCAGTATCT	180
Trib3	GGCTCTCGGCTCCTTTACATC	CCTCGGACTCTGGGATACCG	91
Bmpr1a	TGGCACTGGTATGAAATCAGAC	CAAGGTATCCTCTGGTGCTAAAG	76
Cebpd	CGACTTCAGCGCCTACATTGA	CTAGCGACAGACCCCACAC	171
Spdef	AAGGCAGCATCAGGAGCAATG	CTGTCAATGACGGGACACTG	203
Braf	TGATGCGCTGTCTTCGGAAAT	GCCAGGCTCAAAATCAAACACT	92
Cnr1	AAGTCGATCTTAGACGGCCTT	TCCTAATTTGGATGCCATGTCTC	123
Pik3c2a	TGACAGCCCAAGAGGCTTTG	CCTGGGTGAGCTTTTCTACATC	146
Tbrg3	GTGGTTCTTCCACTCCGGTT	AGCACAGAAAAGCACTGGGA	141
Kcnq1ot1	ACGTGTCTCTCCCCTCTACC	CGCGCACACTAGCATCTTTC	172
Tug1	CTCTGGAGGTGGACGTTTTGT	GTGAGTCGTGTCTCTCTTTTCTC	74
Xist	TCACTCCTGCCTTTCGTGAC	AACAAGTGGGGTGAGCACAA	79
Rnu3a	CACGAGGAGGAGACAGAGTG	GTGTCCTCTCCCTCTCAACC	125
Rn7sk	AGGGTTGATTCGGCTGATCT	CGCAGCTACTCGTATACCCT	159
4930507D05Rik	AGCGAAAGGACCCAGAGAGA	TGCCGCAGAAAGAGTGAGAG	154
